# Sickle cell disease in Africa: an urgent need for longitudinal cohort studies

**DOI:** 10.1016/S2214-109X(19)30364-X

**Published:** 2019-08-23

**Authors:** Ambroise Wonkam, Julie Makani

**Affiliations:** Division of Human Genetics and Institute of Infectious Diseases and Molecular Medicine, Faculty of Health Sciences, University of Cape Town, 7925 Cape Town, South Africa (AW); and Department of Haematology and Blood Transfusion, Muhimbili University of Health and Allied Sciences, Dar-es-Salaam, Tanzania (JM)

Sickle cell disease is caused by a single point mutation (Glu6Val) that promotes polymerisation of haemoglobin S and sickling of erythrocytes. Inflammation, haemolysis, microvascular obstruction, and organ damage characterise the clinical expression of the disease. Environmental and genetic factors influence many pathophysiological aspects of sickle cell disease. It is estimated that 305 800 babies are born each year with the disease worldwide, with nearly 75% of these births in sub-Saharan Africa.^1^ Despite this high incidence, established life-saving public health programmes for sickle cell disease have not been implemented in most African countries. Consequently, childhood mortality due to the disease remains high and estimates suggest that, without interventions, up to 90% of individuals with sickle cell disease in Africa will not reach 18 years of age.^2^

In *The Lancet Global Health*, Sophie Uyoga and colleagues^3^ report the mortality rates of paediatric patients with sickle cell disease in Kenya. This study is one of the few prospective longitudinal investigations of the disease and confirms the high childhood mortality in Africa, in line with previous estimates from modelling approaches.^1^ The study^3^ also confirmed the importance of two key sickle cell disease modifiers—fetal haemoglobin (HbF) and coinheritance of α-thalassaemia— which can influence hospital attendance and survival. These results are similar to previous reports from a prospective cohort in the USA^4^ and cross-sectional studies in Cameroon.^5^ Despite the modest sample size of patients with sickle cell disease who were followed up (n=128), Uyoga and colleagues’ results^3^ support recent efforts to implement newborn screening for the disease,^6^ provision of hydroxycarbamide,^7^ and increased research on genetic modifiers in Africa.^8^ However, this study^3^ did not describe the effect of passive follow-up on major proxies for sickle cell disease severity, such as anaemia, infections, stroke, acute chest syndrome, and acute splenic sequestration. Additionally, the causes of death for the patients in the study and socioeconomic factors that could affect compliance with available medications were not reported. Nevertheless, children who attended the specialised clinic set up by the authors did much better than those who did not attend. This observation highlights the need to urge African governments, and to encourage future prospective projects, to provide the best possible care for patients with sickle cell disease, particularly in resource-poor areas in Africa.

Comprehensive clinical care programmes have reduced premature childhood deaths related to sickle cell disease by 70% in the USA.^9^ Unfortunately, sickle cell disease mortality in adults has not decreased in the last 30 years because of acute and chronic end-organ damage, for example, cardiovascular complications. There remain fundamental gaps in knowledge of the disease, including insufficient understanding of the missing heritability of HbF and the mechanisms controlling switching of fetal to adult haemoglobin, and incomplete identification of disease modifiers to enable develop ment of comprehensive prediction models for patient stratification and targeted interventions. Although sickle cell disease was first described in the medical literature over 100 years ago, until recently, only one drug (hydroxycarbamide) was available to treat the disease. Therefore, there is a major need for research to help develop effective life-long and accessible therapies for patients with sickle cell disease in all parts of the world. These efforts require large longitudinal cohort studies, the most scientifically rigorous methods of understanding environmental, social, and genetic risk factors and health and disease outcomes, and addressing training needs and public health policies. The value of large longitudinal cohorts is shown by the Framingham Heart Study,^10^ which has led to major discoveries in cardiometabolic conditions over the past 70 years. High disease prevalence in sub-Saharan Africa positions this region to lead epidemiological, translational, and clinical research studies on sickle cell disease that cannot be established easily in high-income countries. However, the study by Uyoga and colleagues shows that African nations working in isolation have scarce resources to do such large-scale research.

In response to the need for collaborative efforts, the SickleInAfrica Consortium was established to facilitate multisite, cross-disciplinary, and cross-border sickle cell disease research.^11^ As part of SickleInAfrica efforts, the Sickle Africa Data Coordinating Center (SADaCC), based in South Africa, is building a robust platform to support the activities of its companion, the Sickle Pan African Research Consortium, which is integrating research efforts in Tanzania, Ghana, and Nigeria, with attention to genetic diversity and socioeconomic and environmental factors. As proof of concept, nearly 10 000 patients with sickle cell disease have been enrolled in the SickleInAfrica database over the past 2 years. Before enrolment of these patients, SADaCC had coordinated the development of the most comprehensive knowledge portal for sickle cell disease to date—the first Sickle Cell Disease Ontology (SCDO). The SCDO has enabled the creation of a centralised, electronic haemoglobinopathies database, which includes provision for recruitment tracking systems, data quality and assurance processes, and development and reporting of various research studies designed by SickleInAfrica.

Establishing a multicentre well-coordinated prospective longitudinal sickle cell disease cohort in Africa, which is regionally relevant but has global impact, is crucial to improve our understanding of the pathophysiology of the disease and investigate new routes for novel therapeutic interventions with the aim of reducing the substantial disease burden.

## References

[1] Piel FB, Hay SI, Gupta S, Weatherall DJ, Williams TN. Global burden of sickle cell anaemia in children under five, 2010–2050: modelling based on demographics, excess mortality, and interventions. PLoS Med 2013; 10: e1001484. 23874164 10.1371/journal.pmed.1001484 PMC3712914

[2] Grosse SD, Odame I, Atrash HK, Amendah DD, Piel FB, Williams TN. Sickle cell disease in Africa: a neglected cause of early childhood mortality. Am J Prev Med 2011; 41 (6 suppl 4): S398–405. 22099364 10.1016/j.amepre.2011.09.013 PMC3708126

[3] Uyoga S, Macharia AW, Mochamah G, The epidemiology of sickle cell disease in children recruited in infancy in Kilifi, Kenya: a prospective cohort study. Lancet Glob Health 2019; published online Aug 23. 10.1016/S2214-109X(19)30328-6. PMC7024980 31451441

[4] Platt OS, Brambilla DJ, Rosse WF, Mortality in sickle cell disease. Life expectancy and risk factors for early death. N Engl J Med 1994; 330: 1639–44. 7993409 10.1056/NEJM199406093302303

[5] Wonkam A, Rumaney MB, Ngo Bitoungui VJ, Vorster AA, Ramesar R, Ngogang J. Coinheritance of sickle cell anemia and α-thalassemia delays disease onset and could improve survival in Cameroonian’s patients (sub-Saharan Africa). Am J Hematol 2014; 89: 664–65 10.1002/ajh.23711 24639123

[6] Nkya S, Mtei L, Soka D, Newborn screening for sickle cell disease: an innovative pilot program to improve child survival in Dar es Salaam, Tanzania. Int Health 2019; published online May 30. DOI:10.1093/inthealth/ihz028. PMC7967808 31145786

[7] Tshilolo L, Tomlinson G, Williams TN, Hydroxyurea for children with sickle cell anemia in sub-Saharan Africa. N Engl J Med 2019; 380: 121–31. 30501550 10.1056/NEJMoa1813598 PMC6454575

[8] Geard A, Pule GD, Chetcha Chemegni B, Clinical and genetic predictors of renal dysfunctions in sickle cell anaemia in Cameroon. Br J Haematol 2017; 178: 629–39. 28466968 10.1111/bjh.14724 PMC5660286

[9] Chaturvedi S, DeBaun MR. Evolution of sickle cell disease from a life-threatening disease of children to a chronic disease of adults: the last 40 years. Am J Hematol 2016; 91: 5–14. 26547630 10.1002/ajh.24235

[10] Long MT, Fox CS. The Framingham Heart Study—67 years of discovery in metabolic disease. Nat Rev Endocrinol 2016; 12: 177–83. 26775764 10.1038/nrendo.2015.226

[11] Makani J, Ofori-Acquah SF, Tluway F, Mulder N, Wonkam A. Sickle cell disease: tipping the balance of genomic research to catalyse discoveries in Africa. Lancet 2017; 389: 2355–58. 28635598 10.1016/S0140-6736(17)31615-X PMC5612389

